# Veterinarian–pharmacist collaboration in veterinary medicine: Roles, practices, and perceptions—A scoping review

**DOI:** 10.1371/journal.pone.0355233

**Published:** 2026-07-31

**Authors:** Dan Kambayashi, Shota Suzuki, Masayoshi Hirohara

**Affiliations:** 1 Laboratory of Pharmacy Practice, Center for Education and Research on Clinical Pharmacy, Showa Pharmaceutical University, Machida, Tokyo, Japan; 2 Institute for Clinical and Translational Science, Nara Medical University Hospital, Kashihara, Japan; 3 Department of Social & Community Pharmacy, School of Pharmaceutical Sciences, Wakayama Medical University, Wakayama, Japan; Endeavour College of Natural Health, AUSTRALIA

## Abstract

**Background:**

Pharmacists are expanding their participation in veterinary healthcare teams and assuming roles beyond traditional dispensing duties. However, the scope of these collaborative practices and the degree of mutual recognition vary substantially across regions.

**Objectives:**

This scoping review aimed to organize the existing literature on collaboration between veterinarians and pharmacists, clarifying current roles, practical applications, and professional perceptions within the veterinary field.

**Methods:**

A scoping review was conducted to examine the roles, practices, and perceptions associated with veterinarian–pharmacist collaboration in veterinary medicine and related fields. Two researchers independently searched PubMed, Web of Science, the Cochrane Library, and Ichushi-Web, and additionally screened Google Scholar to identify gray literature (from database inception to August 2025, in English or Japanese). Records were independently screened at the title/abstract and full-text levels using predefined eligibility criteria, and relevant studies were identified.

**Results:**

The search yielded 239 records, of which 16 studies published between 2007 and 2024 were included. Studies were conducted primarily in the United States (n = 7), New Zealand (n = 3), and Japan (n = 3); one study collected data from both Japan and Taiwan. Most studies employed cross-sectional survey designs. Pharmacist roles most frequently involved compounding and dispensing for animal patients (62.5%), followed by drug information (DI) and consultation (37.5%), inventory and supply management (25.0%), client education (18.8%), and safety and exposure control (12.5%).

**Conclusions:**

This scoping review demonstrates that veterinarian–pharmacist collaboration is described within a limited and regionally variable evidence base, with pharmacists most often contributing through compounding/dispensing and drug information support. Sustained and scalable implementation will require improved mutual understanding of professional roles, strengthened veterinary-specific education for pharmacists, and more robust empirical research to inform collaborative practice models.

## Introduction

In recent years, veterinary medicine has undergone a remarkable transformation, mirroring the increasing sophistication and complexity of human healthcare [1,2]. This evolution reflects not only technological progress but also a growing recognition of companion animals as integral members of families and communities [3]. Because companion animals and other veterinary patients are increasingly perceived as family, their health has become inextricably linked to the physical and emotional well-being of their owners [4,5]. The demand for high-quality, specialized veterinary care has concurrently increased, placing unprecedented responsibility on veterinarians to manage complex drug regimens similar to those encountered in human medicine [6].

To address these growing challenges, the broader integration of pharmacists into the veterinary healthcare team represents a logical and necessary evolution. Although veterinary clinical pharmacologists also possess comparable specialized skills, pharmacists remain an underutilized resource in veterinary practice. Just as interprofessional collaboration has become the gold standard in human healthcare to ensure medication safety [7,8], a comparable partnership in veterinary medicine holds substantial promise. By leveraging pharmacists’ expertise in pharmacology alongside veterinarians’ diagnostic acumen, the healthcare team can optimize clinical outcomes and reduce medication-related errors [9]. Moreover, this collaboration extends beyond the clinic by supporting owners in their ability to care for their animals, thereby preserving the human–animal bond. Ultimately, establishment of a robust veterinarian–pharmacist partnership creates a synergistic environment in which veterinarians, pharmacists, animal patients, and their owners all derive mutual benefit [10].

Despite these apparent advantages, practical implementation of veterinarian–pharmacist collaboration remains fragmented and inconsistent [11]. Unlike the well-established interprofessional models found in human healthcare, the veterinary–pharmacist interface lacks a standardized framework or clearly defined roles [12]. Although instances of collaboration do exist, they often occur in isolation, driven by both a structural disconnect and a mutual lack of understanding between the two professions regarding each other's scopes of practice and specialized skills [13]. As a result, how veterinarians and pharmacists actually work together remains largely unexplored and insufficiently documented in a systematic manner.

The purpose of this scoping review is to map the existing literature on veterinarian–pharmacist collaboration. By clarifying the current landscape of collaborative practices, identifying key characteristics, and highlighting gaps in the literature, this review aims to provide a foundational roadmap for fostering a mutually beneficial partnership that enhances outcomes for veterinarians, pharmacists, animal patients, and owners alike.

## Methods

### Study design and protocol

The review protocol was prepared using a template developed by the Joanna Briggs Institute [14]. The conduct and reporting of this scoping review were guided by the Preferred Reporting Items for Systematic Reviews and Meta-Analyses extension for Scoping Reviews guidelines [15]. No protocol was registered for this review.

### Eligibility criteria

The eligibility criteria were developed using the Population, Concept, and Context (PCC) framework. The population comprised veterinarians and pharmacists. The concept was defined as collaborative practice between veterinarians and pharmacists, as well as activities intended to establish, promote, or evaluate such collaboration. The context encompassed animal health, veterinary medicine, public health, and One Health–related domains, including clinical practice, research, and community-based activities.

### Inclusion criteria

Articles were included if they involved both veterinarians and pharmacists in roles consistent with their respective professional scopes of practice and reported collaborative activities, practice, or research undertaken jointly by veterinarians and pharmacists.

### Exclusion criteria

Articles were excluded if only one of the two professions, either a veterinarian or a pharmacist, was involved or mentioned, or if the nature of veterinarian–pharmacist collaboration was not described in sufficient detail. Articles were also excluded when activities were primarily student-led and lacked educational or practice-based involvement of veterinarians and/or pharmacists, when content was limited to recommendations or commentary not grounded in clinical data or empirical survey data, or when the article was not relevant to veterinary practice or public health practice. In addition, articles not written in English or Japanese, as well as those for which the full text could not be retrieved or confirmed, were excluded.

### Information sources and search strategy

Two reviewers (DK and SS) independently searched for eligible studies published in PubMed, Web of Science, the Cochrane Library, and Ichushi-Web (Japan Medical Abstracts Society). While CAB Abstracts is widely recognized as a premier database for veterinary literature, we utilized Web of Science and PubMed to capture literature at the multidisciplinary intersection of human biomedical sciences, pharmacy, and veterinary medicine. In addition, Google Scholar was searched to identify gray literature. No restrictions were applied to publication date (search from database inception). The search was limited to articles written in English or Japanese. The final search was conducted on 30 August 2025. Search strategies were tailored to each database. The following keywords were used: “pharmacist” AND “veterinarian,” using English terms and Japanese equivalents as appropriate for each source. Detailed search strategies for each database are provided in the Supplementary Material (S1 Appendix). The search strategy was developed exclusively by the authors; a medical librarian or information specialist was not consulted during its design or execution.

### Selection of sources of evidence

All retrieved citations were uploaded to Rayyan [16], a web-based collaboration platform designed specifically to streamline the screening process for systematic and scoping reviews. Within this platform, duplicate records were identified and removed prior to screening. In the first screening stage, studies retrieved from PubMed, Web of Science, the Cochrane Library, and Ichushi-Web were assessed based on titles and abstracts, whereas records identified through Google Scholar were evaluated using titles and document types to determine eligibility. Full-text articles that met the inclusion criteria at this stage underwent subsequent full-text screening.

Both screening stages were performed independently by two reviewers (DK and SS). Any disagreements were resolved through discussion, with arbitration by a third reviewer (MH) when necessary.

### Data charting process and data items

Key bibliographic data (title, authors, publication year, country, language, research approach, and main outcomes) were charted for all eligible studies. Each study was then grouped into one of five themes. These themes were developed through an iterative process; initial broad categories were established *a priori* based on the authors’ expertise and research objectives and were subsequently refined and finalized during the data extraction phase. Additional data were extracted as appropriate.

All extracted data were compiled in Microsoft Excel (S1 Dataset). Contributions from each researcher were examined and discussed by the full research team to reach consensus.

### Synthesis of results

In line with the aims of this review, included studies were classified according to their study designs and the nature of collaboration between veterinarians and pharmacists. In addition, key considerations for future research were identified and assessed.

### Ethics statement

Institutional review board approval and patient consent were not required because of the review nature of this study.

## Results

### Selection of sources of evidence

**Fig 1:**
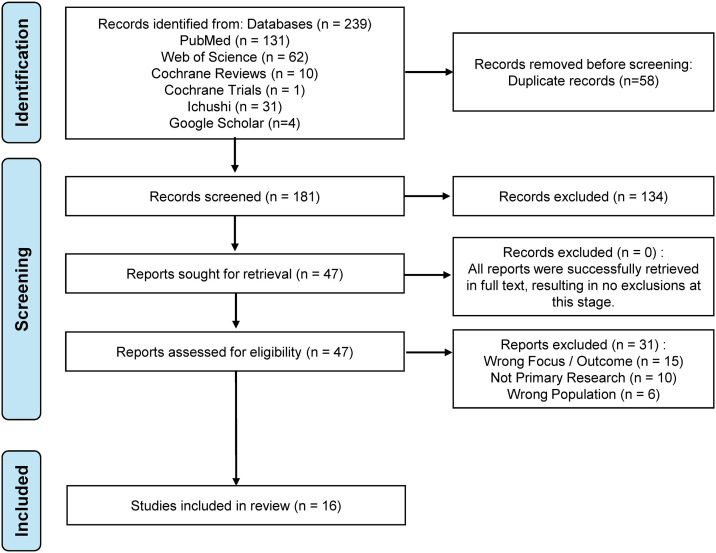
PRISMA flow diagram of article identification and inclusion in the review.

In total, 239 records were retrieved through database searches, including PubMed (n = 131), Web of Science (n = 62), Cochrane Reviews (n = 10), Cochrane Trials (n = 1), Ichushi-Web (n = 31), and Google Scholar (n = 4). After removal of 58 duplicate records, 181 records were retained for screening. During the initial screening phase, 134 records were excluded based on titles, abstracts, and publication types. Full-text assessment was subsequently conducted for the remaining reports, resulting in the exclusion of an additional 31 records. Consequently, 16 studies [10,12,17–30] were included in the final review (Fig 1).

### Characteristics of the included studies

**Fig 2:**
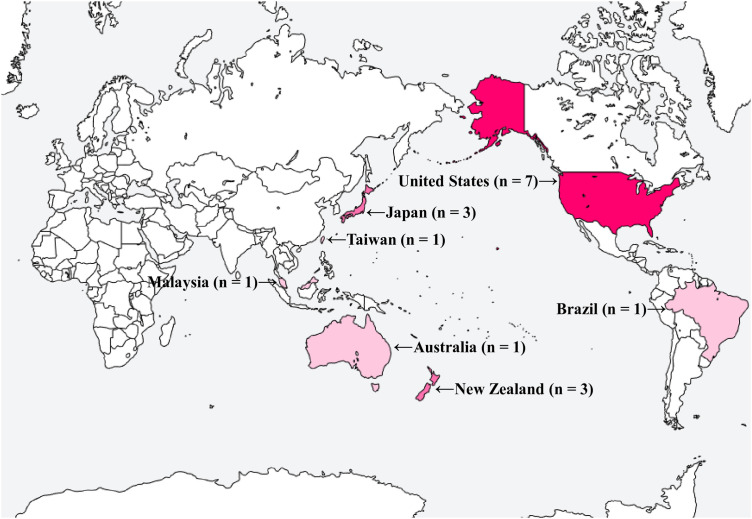
Geographic distribution of the included studies (n = 16). The United States was the largest contributor (n = 7), followed by New Zealand (n = 3) and Japan (n = 3). Other contributing regions include Australia, Brazil, Malaysia, and Taiwan. Darker shading indicates a higher frequency of reports, illustrating that veterinarian–pharmacist collaboration is a global phenomenon evolving across diverse regulatory environments. Note: One study collected data from both Japan and Taiwan. The base map was obtained from Freemap.jp (https://n.freemap.jp/), which provides vector maps free of copyright restrictions.

Table 1 summarizes the characteristics of the 16 included studies. Publications spanned from 2007 to 2024 and were conducted across several countries, most frequently in the United States (n = 7) [12,17,19,22–25], followed by New Zealand (n = 3) [20,21,26] and Japan (n = 3) [10,27,28]. One study was conducted across both Japan and Taiwan. Additional studies were conducted in Australia [18], Taiwan [27], Malaysia [30], and Brazil [29]. Most studies were published in English, although two were reported in Japanese [27,28] (Fig 2).

**Table 1:** Characteristics of included articles (n =  16).

Author	Year	Country	Language	Study design	Participants	Target species	Setting	Category	Main focus of the study
Simpson [17]	2007	United States	English	Case report	Cats	Companion animals (cats)	Veterinary hospital	Practical, Clinical, and Activity Reports	In feline behavioral therapy, pharmacists play an essential role in supporting owner adherence and animal welfare by applying pharmaceutical expertise to provide medications in easy-to-administer forms, such as flavored liquids or transdermal patches.
Ceresia et al. [12]	2009	United States and Canada (panelists’ residence)	English	Delphi method	Panel of 143 experts (veterinarians, veterinary hospital pharmacists, pharmaceutical educators, and others)	General (small animals, large animals, wildlife/exotic animals)	University / expert network	Research and Studies	A consensus was reached on a broad scope of responsibilities for veterinary pharmacists, including dispensing, prescription design, pharmacokinetic monitoring, education, and regulatory compliance.
Haywood et al. [18]	2009	Australia	English	Qualitative research	Veterinarians at a wildlife theme park (3 individuals)	Exotic animals, wildlife animals	Zoo	Research and Studies	This qualitative study identified practical challenges in administering medications to wild animals and demonstrated that pharmacist-led dispensing can effectively address these challenges and improve treatment outcomes.
Meadows and Muller [19]	2010	United States	English	Expert opinion	Veterinarian, pharmacist	Companion animals (cats)	Veterinary hospital	Practical, Clinical, and Activity Reports	Successful CKD treatment in cats relies on pharmacists reformulating human medications into palatable and practical dosage forms (e.g., liquids or suspensions), reducing the burden on owners and supporting continued treatment while maintaining the cat’s quality of life.
McDowell et al. [20]	2011	New Zealand	English	Qualitative research	Urban veterinarians (5 animal hospitals)	Companion animals	Veterinary hospital	Research and Studies	Given veterinarians’ monopoly on dispensing and pharmaceutical sales, collaboration with pharmacists—particularly through their expertise in human medicines and procurement systems—can improve treatment safety, reduce costs for animal owners, and enhance inventory management and regulatory compliance.
Gargiulo et al. [21]	2013	New Zealand	English	Qualitative research / cross-sectional study	Veterinarians (99 respondents), animal facility and pharmacy personnel	Dogs, cats, cows, and others	Zoo / veterinary hospital	Research and Studies	Veterinarians frequently require compounded medications but face challenges related to stability and formulation techniques. Leveraging pharmacists’ expertise in biopharmaceutics can address these issues and enable safer, more effective treatment for animal patients.
Pruitt [22]	2013	United States	English	Case report	Horses (racehorses, Irish sport horses)	Horses	Veterinary hospital	Practical, Clinical, and Activity Reports	For large animals such as horses, where administering standard human medications is challenging, pharmacists can significantly improve treatment adherence by compounding customized suspensions tailored to animal preferences (e.g., peppermint flavor).
Root Kustritz et al. [23]	2013	United States	English	Cross-sectional study	Veterinarians who are members of the American Veterinary Medical Association (769 respondents)	Small animals, large animals, exotic animals, and others	University / government and administrative agencies / businesses	Research and Studies	Veterinarians regularly collaborate with pharmacists, often on a monthly to weekly basis, particularly to verify specialized pharmaceutical information such as drug interactions, contraindications, and species-specific metabolic differences.
Bennett et al. [24]	2018	United States	English	Cross-sectional study	Animal owners (118 individuals), veterinarians (15 individuals)	Companion animals	Pharmacy / veterinary hospital	Research and Studies	While many animal owners and veterinarians express interest in veterinary compounding at local pharmacies (e.g., flavor modification), veterinarians report concerns regarding pharmacists’ level of veterinary-specific knowledge.
Fredrickson et al. [25]	2020	United States	English	Cross-sectional study	Pharmacists (357 individuals), veterinarians (232 individuals)	Companion animals	Pharmacy / veterinary hospital	Research and Studies	Although both professions recognize the value of collaboration and report frequent practical interactions, veterinarians remain hesitant to accept pharmacists as clinical counselors, instead emphasizing their role in compounding.
Besley et al. [26]	2023	New Zealand	English	Qualitative / cross-sectional	Veterinarians at zoos and wildlife parks (8 responses, 4 interviews)	Wildlife animals, zoo animals (including exotic animals)	Zoo	Research and Studies	Veterinarians highly value pharmacists as specialists in compounding and dispensing, particularly when commercially available medications are inadequate, and recognize their contributions to maintaining wildlife health.
Shimizu et al. [27]	2023	Japan / Taiwan	Japanese	Cross-sectional study	Animal owners (118 in Japan, 133 in Taiwan), veterinarians at animal hospitals (56 facilities)	Companion animals	Pharmacy / veterinary hospital	Research and Studies	Pharmacists can compensate for veterinarians’ limitations in inventory management and pharmacological knowledge by supplying herbal medicines and developing improved dosage forms, thereby expanding treatment options through information sharing.
Otsuka and Asai [28]	2023	Japan	Japanese	Activity report / cross-sectional study	Veterinarians working at animal hospitals (15 individuals), veterinary nurses (20 individuals)	Companion animals	Veterinary hospital	Practical, Clinical, and Activity Reports	Establishing optimized inventory management is identified as the first step for pharmacists working in veterinary hospitals, forming the foundation for team-based care in which veterinarians act as clinical consultation partners and nurses as specialists in owner education.
Ferreira et al. [29]	2024	Brazil	English	Cross-sectional study	Veterinarians (62 individuals), pharmacists (45 individuals), and others totaling 108 individuals	Companion animals	Veterinary hospital	Research and Studies	Veterinary facilities in which pharmacists oversee the preparation of anticancer (cytotoxic) agents demonstrate safer infrastructure and experience fewer occupational accidents compared with facilities led by veterinarians.
Konno et al. [10]	2024	Japan	English	Cross-sectional study	Pharmacy and drugstore staff (324 individuals), veterinary hospital staff (217 individuals)	Companion animals	Pharmacy / veterinary hospital	Research and Studies	Japanese veterinarians expect pharmacists to contribute beyond dispensing, particularly through drug information provision and inventory management, yet a gap in mutual understanding between the two professions persists.
Paneerselvam et al. [30]	2024	Malaysia	English	Cross-sectional study	Veterinarians at private animal hospitals (40 individuals)	Companion animals	Veterinary hospital	Research and Studies	More than half of Malaysian veterinarians reported positive attitudes toward collaboration with pharmacists, particularly valuing contributions to dispensing techniques and inventory management efficiency, although trust in pharmacists’ expertise remains essential for future expansion.

Abbreviations: CKD, chronic kidney disease.

Regarding study design, the majority employed cross-sectional survey methods [10,21,23–30], while qualitative studies, review articles, and expert consensus-based research were also represented. Participants included pharmacists, veterinarians, and animal owners, reflecting multiple perspectives on interprofessional collaboration in veterinary pharmacotherapy. Studies were conducted in diverse settings, including community pharmacies (n = 4) [10,24,25,27], veterinary hospitals (n = 12) [10,17,19–22,24,25,27–30], zoos (n = 3) [18,21,26], and academic or expert networks (n = 2) [12,23]. Overall, the literature highlighted pharmacists’ contributions to veterinary care, such as compounding and dispensing for animal patients, providing drug information to veterinarians and owners, supporting medication safety, and identifying barriers and opportunities to expand pharmacist–veterinarian collaboration.

### Pharmacist roles in veterinary medicine

Table 2 summarizes the pharmacist roles reported in the included studies across different countries. Overall, the most frequently described role was compounding and dispensing, identified in 10 of the 16 studies (62.5%). This activity was most commonly reported in studies from the United States (n = 6) [12,17,19,22,24,25] and New Zealand (n = 2) [21,26], with additional reports from Australia (n = 1) [18] and Brazil (n = 1) [29]. Drug information and consultation were described in 6 studies (37.5%), including those conducted in the United States (n = 3) [12,19,23], Japan (n = 1) [10], Japan/Taiwan (n = 1) [27], and Malaysia (n = 1) [30]. Inventory and supply management were mentioned in 4 studies (25.0%), most frequently in Japan (n = 2) [10,28], followed by New Zealand (n = 1) [20] and the United States (n = 1) [12]. Safety and exposure control were identified in only 2 studies (12.5%), conducted in Brazil (n = 1) [29] and the United States (n = 1) [12]. Finally, client education was reported in 3 studies (18.8%), including studies from Japan (n = 1) [28], Japan/Taiwan (n = 1) [27], and the United States (n = 1) [12].

**Table 2:** Role of pharmacists in veterinary medicine when collaborating with veterinarians.

Author (Country)	Compounding	DI/Consultation	Inventory/Supply	Safety/Exposure	Client Education
Simpson [17] (USA)	●				
Ceresia et al. [12] (USA)	●	●	●	●	●
Haywood et al. [18] (AUS)	●				
Meadows and Muller [19] (USA)	●	●			
McDowell et al. [20] (NZ)			●		
Gargiulo et al. [21] (NZ)	●				
Pruitt [22] (USA)	●				
Root Kustritz et al. [23] (USA)		●			
Bennett et al. [24] (USA)	●				
Fredrickson et al. [25] (USA)	●				
Besley et al. [26] (NZ)	●				
Shimizu et al. [27] (JPN/TWN)		●			●
Otsuka and Asai [28] (JPN)			●		●
Ferreira et al. [29] (BRA)	●			●	
Konno et al. [10] (JPN)		●	●		
Paneerselvam et al. [30] (MYS)		●			

Abbreviations: DI, drug information; USA, United States of America; AUS, Australia; NZ, New Zealand; JPN, Japan; TWN, Taiwan; BRA, Brazil; MYS, Malaysia

## Discussion

This scoping review mapped the landscape of veterinarian–pharmacist collaboration based on 16 included studies. The analysis indicates that while the pharmacist’s role in veterinary medicine is expanding beyond traditional dispensing, the nature of this collaboration varies considerably by region and practice setting. Distinct regional patterns were observed: a focus on compounding services appears dominant in Western regions (United States, Oceania), whereas emphasis on inventory management and hospital safety is more frequently reported in Asia and South America. Despite mutual recognition of the potential benefits of interprofessional teamwork, a consistent barrier identified across multiple studies is veterinarians’ concern regarding pharmacists’ limited training in species-specific physiology. This perceived “knowledge gap” appears to be a major factor limiting progression from administrative interactions to more integrated clinical collaboration.

### Barriers to collaboration

Hesitation to delegate clinical tasks to pharmacists is rooted largely in concerns about professional competency related to non-human species. Surveys from the United States and Malaysia indicate that while veterinarians value logistical support, they remain cautious about pharmacists providing clinical advice because of the risks associated with species-specific toxicology and metabolic differences [24,25,30]. Veterinarians often view pharmacists as experts in human pharmacotherapy who may inadvertently apply human medical logic to animal patients. Indeed, real-world reports and surveys from professional organizations, such as the American Veterinary Medical Association, consistently document these practical barriers [31–33]. Recurrent issues include unauthorized therapeutic substitutions and unapproved dosage alterations by community pharmacies, which in some instances have led to adverse patient outcomes [31–33]. Furthermore, significant regulatory variations and a lack of validated compounding formulas for animal patients continue to pose serious risks of toxicity and therapeutic failure [34]. While these clinical safety concerns highlight the critical need for collaborative dialogue, interprofessional integration is further hindered by a fundamental disconnect in role perception. In Japan, for example, pharmacists express a desire to contribute more directly to animal care, yet their envisioned roles do not always align with the practical needs of veterinarians, who prioritize support in inventory management and drug information rather than general dispensing [10]. In addition, the need for pharmacy education regarding veterinary medicine has been increasingly reported in multiple countries [35–37]. Collectively, these findings suggest that foundational education in veterinary pharmacotherapy is a prerequisite for pharmacists to gain the professional trust required for deeper integration into veterinary healthcare teams.

### Evolution of roles

Despite these barriers, the literature demonstrates a diversification of pharmacists’ contributions. Historically, particularly in Western contexts, the primary value of pharmacists in veterinary medicine has been technical, most notably in compounding medications to address administration challenges. Several studies highlight how pharmacists facilitate therapeutic adherence by modifying dosage forms, such as creating transdermal gels or flavoring medications for animal patients with chronic conditions [17,19,22]. More recent reports, however, suggest an expanding scope in which pharmacists are increasingly valued for their management capabilities. In animal hospital settings, pharmacists are beginning to alleviate administrative burdens by optimizing inventory control and managing drug supplies [10,28]. Moreover, in high-risk environments such as veterinary oncology, pharmacists are emerging as key contributors to occupational safety. Evidence from Brazil indicates that pharmacist-led management of antineoplastic agents is associated with improved adherence to biosafety protocols, thereby reducing hazardous exposure among veterinary staff [29]. This shift from “product-centered” to “system-centered” contributions reflects a notable maturation of the pharmacist’s role within veterinary medicine.

### Regional variations in collaboration models

This review found that collaboration models are strongly shaped by local regulatory environments and medical cultures. In North America and Oceania (United States, New Zealand, Australia), collaboration is predominantly technique-driven, centered on compounding to address gaps in approved veterinary products or suitable dosage forms [12,17–19,21,22,24–26]. By contrast, a management- and information-driven model appears more prominent in parts of Asia. In countries such as Japan and Malaysia, where in-house dispensing by veterinarians is common, pharmacists often enter the care team through drug information services, inventory management, or complementary medicine [10,30]. For instance, in Japan and Taiwan, pharmacists play a distinctive role in advising owners on Kampo (traditional herbal medicine), effectively bridging human traditional medicine and veterinary applications [27].

### Importance of non-English evidence

An important finding of this review is that Japan is a significant contributor to the literature (n = 3), comparable to New Zealand and second only to the United States. This suggests substantial interest and the development of distinct practice models in non-Western contexts. However, valuable insights—such as pharmacist involvement in Kampo medicine or detailed inventory systems reported in Japanese-language studies [27,28]—remain largely inaccessible to the broader international community because of language barriers. Exclusion of non-English articles from systematic searches risks overlooking innovative, locally developed models that may address shared global challenges. Given recent advances in neural machine translation technologies, future researchers and practitioners should actively seek out and translate relevant non-English literature, including databases such as Ichushi-Web. Addressing this linguistic barrier is essential to capturing the full diversity and potential of global veterinarian–pharmacist collaboration.

### Value of pharmacists in highly specialized contexts

Pharmacists’ expertise appears to be most readily accepted in specialized clinical environments where standard protocols are insufficient. In zoological and wildlife settings, extreme species diversity creates complex therapeutic challenges that require advanced pharmaceutical reasoning related to pharmacokinetics (i.e., how an animal’s body absorbs, distributes, metabolizes, and excretes drugs) and formulation (i.e., the process of preparing customized dosage forms to suit species-specific anatomical and physiological needs). In these contexts, pharmacists are utilized not merely as dispensers but as problem-solvers within the veterinary team [18,26]. Similarly, in veterinary university hospitals and specialty centers, the complexity of care necessitates a level of pharmaceutical oversight—such as sterile compounding and safety management—that may exceed the needs of general practice [12,29].

### Limitations

This scoping review has several limitations. First, many included studies relied on cross-sectional surveys with relatively small sample sizes or low response rates, limiting generalizability. Some studies were confined to specific geographic regions or states, potentially failing to capture the full range of practice models within a country. Second, selection bias is possible because respondents may have had a pre-existing interest in or favorable attitudes toward interprofessional collaboration. Third, most evidence was perception-based, relying on surveys and interviews rather than objective clinical outcomes. There remains a notable lack of quantitative research assessing the direct impact of pharmacist involvement on therapeutic outcomes or adverse event reduction in veterinary settings. Finally, our search strategy relied on the aforementioned databases, particularly PubMed and Web of Science. Although Web of Science offers extensive multidisciplinary coverage, including veterinary journals, we did not search CAB Abstracts, which has been shown to have the broadest coverage of veterinary literature [38]. Consequently, it is possible that some relevant articles published in exclusively veterinary or regional agricultural journals were omitted. Future systematic reviews on this topic should incorporate CAB Abstracts to ensure maximum comprehensiveness from the veterinary perspective. Despite these limitations, this review represents the first comprehensive synthesis of the global literature on veterinarian–pharmacist collaboration and provides a critical baseline for understanding this evolving professional relationship.

### Future implications

Advancing the pharmacist’s role from an external service provider to an integrated partner in veterinary healthcare requires progress in several areas. First, specialized education is essential. Pharmacy curricula and continuing education programs must more systematically incorporate veterinary-specific physiology, pharmacology, and veterinary healthcare systems to address the knowledge gap that currently undermines trust [10,12]. Furthermore, expanding practical training opportunities—such as veterinary pharmacy residencies, externships, and elective courses—provides essential pathways for pharmacists to integrate successfully into veterinary practice environments [39,40]. Second, sustained interprofessional dialogue is needed. Clarifying mutual expectations and limitations—potentially through interprofessional education initiatives—may reduce professional friction and foster collaborative cultures [23]. Finally, stronger evidence is required. Future research should move beyond perception-based surveys to evaluate measurable clinical, safety, and economic outcomes of pharmacist involvement, including improvements in occupational safety compliance and hospital management efficiency. To facilitate meaningful quantitative research in the future, a phased approach may be beneficial. Given that the presence of pharmacists within veterinary hospitals is currently extremely rare, an important initial step may be the development of veterinarian–pharmacist collaborative community models that connect local clinics with community pharmacies. Concurrently, the establishment of standardized veterinary dispensing guidelines may help promote consistency and species-specific safety in pharmaceutical care. Once these foundational frameworks are in place, they may provide the infrastructure and structured data needed to evaluate the impact of pharmacist involvement on clinical outcomes, such as medication adherence, dispensing errors, owner satisfaction, and measurable physiological outcomes.

## Conclusions

Pharmacists are expanding their role in veterinary medicine, evolving from traditional dispensing functions to active contributors to clinical care and medication safety. Although the scope and nature of collaboration vary globally, a persistent barrier remains veterinarians’ concern regarding pharmacists’ limited training in animal physiology. Addressing this gap through specialized veterinary education for pharmacists is a critical step toward establishing interprofessional trust. Strengthening this partnership has the potential to enhance occupational safety and practice efficiency for veterinarians while optimizing therapeutic outcomes for animal patients.

## Supporting information

S1 Checklist PRISMA-ScR Checklist. (PDF)

S1 Appendix Search strategies for all databases. (DOCX)

S1 Dataset Minimal data set of the scoping review. (XLSX)
