# Supplementary material for: Veterinarian–pharmacist collaboration in veterinary medicine: Roles, practices, and perceptions—A scoping review
Source: PLoS One. 2026 Jul 31;21(7):e0355233. doi: 10.1371/journal.pone.0355233 (PMC13426993; doi:10.1371/journal.pone.0355233)
Supplement: S1 Appendix — (DOCX) [file pone.0355233.s002.docx]

**S1 Appendix.**

Databases searched and search strategy (Search date: August 30, 2025).

| **Database** | **Combination of terms** |
| --- | --- |
| PubMed | ("pharmacist s"[All Fields] OR "pharmacists"[MeSH Terms] OR "pharmacists"[All Fields] OR "pharmacist"[All Fields]) AND ("veterinarian s"[All Fields] OR "veterinarians"[MeSH Terms] OR "veterinarians"[All Fields] OR "veterinarian"[All Fields]) |
| Web of Science | (Pharmacist or 薬剤師) and (Veterinarian or 獣医師) |
| Cochrane Library | (Pharmacist):ti,ab,kw AND (veterinarian):ti,ab,kw |
| Ichushi-Web | (薬剤師 OR pharmacist) AND (獣医師 OR veterinarian) |
| Google Scholar | allintitle: (薬剤師 OR pharmacist) AND (獣医師 OR veterinarian) |

Note: "薬剤師" and "獣医師" are Japanese terms for "Pharmacist" and "Veterinarian", respectively.
